# Pathogenic Connexin-31 Forms Constitutively Active Hemichannels to Promote Necrotic Cell Death

**DOI:** 10.1371/journal.pone.0032531

**Published:** 2012-02-29

**Authors:** Jingwei Chi, Li Li, Mujun Liu, Jieqiong Tan, Chengyuan Tang, Qian Pan, Danling Wang, Zhuohua Zhang

**Affiliations:** 1 The State Key Laboratory of Medical Genetics, Xiangya Medical School, Central South University, Changsha, Hunan, China; 2 School of Biological Science and Technology, Central South University, Changsha, Hunan, China; Emory University, United States of America

## Abstract

Mutations in Connexin-31 (Cx31) are associated with multiple human diseases including erythrokeratodermia variabilis (EKV). The molecular action of Cx31 pathogenic mutants remains largely elusive. We report here that expression of EKV pathogenic mutant Cx31R42P induces cell death with necrotic characteristics. Inhibition of hemichannel activity by a connexin hemichannel inhibitor or high extracellular calcium suppresses Cx31R42P-induced cell death. Expression of Cx31R42P induces ER stress resulting in reactive oxygen species (ROS) production, in turn, to regulate gating of Cx31R42P hemichannels and Cx31R42P induced cell death. Moreover, Cx31R42P hemichannels play an important role in mediating ATP release from the cell. In contrast, no hemichannel activity was detected with cells expressing wildtype Cx31. Together, the results suggest that Cx31R42P forms constitutively active hemichannels to promote necrotic cell death. The Cx31R42P active hemichannels are likely resulted by an ER stress mediated ROS overproduction. The study identifies a mechanism of EKV pathogenesis induced by a Cx31 mutant and provides a new avenue for potential treatment strategy of the disease.

## Introduction

Connexins form gap junction channels between adjacent cells to mediate direct exchange of small cytoplasmic molecules and metabolites less than 1KD. They can also form hemichannels on unopposed plasma membrane and allow the passage of small molecules, such as ATP and glutamine [1]–[2]. Gating of connexin hemichannels is not well understood. However, the hemichannel open probability is regulated by membrane depolarization, extracellular calcium, metabolism inhibition and oxidative stress [3]–[6]. Excessive hemichannel opening is considered to be responsible for cell death induced by Keratitis-ichthyosis-deafness syndrome (KIDS) associated Cx26 mutants [7]–[10], hidrotic ectodermal dysplasia (HED) related Cx30 mutants [11] and X-linked Charcot-Marie-Tooth (CMTX) associated Cx32 mutants [12].

Mutations in Cx31 are associated with multiple diseases, including hearing impairment [13], erythrokeratodermia variabilis (EKV) [14]–[15], and peripheral neuropathy [16]. Previous studies suggest that these pathogenic mutants are abnormal in trafficking [17]–[19]. EKV is a rare hereditary skin disease characterized by fixed hyperkeratotic plaques and transient erythema [14], [20]. Both EKV and hearing loss associated mutations can induce ER stress when they are transiently expressed in cells [19], [21]. However, only EKV-associated mutations are observed to cause cell death [17], [19], [21]–[22].

To investigate functional mechanism of pathogenic Cx31 mutants, we established cell lines stably expressing wildtype Cx31 and EKV associated mutation R42P. Cx31R42P stable cell lines expressing mutant protein at neglective level when cultured at 37°C. However, the amount of mutant proteins increased and cell death phenotype was observed when cells were grown at 26°C. The potential mechanism for Cx31R42P promoting cell death is that the mutant protein induces ER stress resulting in overproduction of reactive oxygen species (ROS). In turn, excessive ROS promote Cx31R42P hemichannels opening, leading to cell death.

## Results

### Expression of Cx31R42P induces necrotic cell death

**Figure 1:**
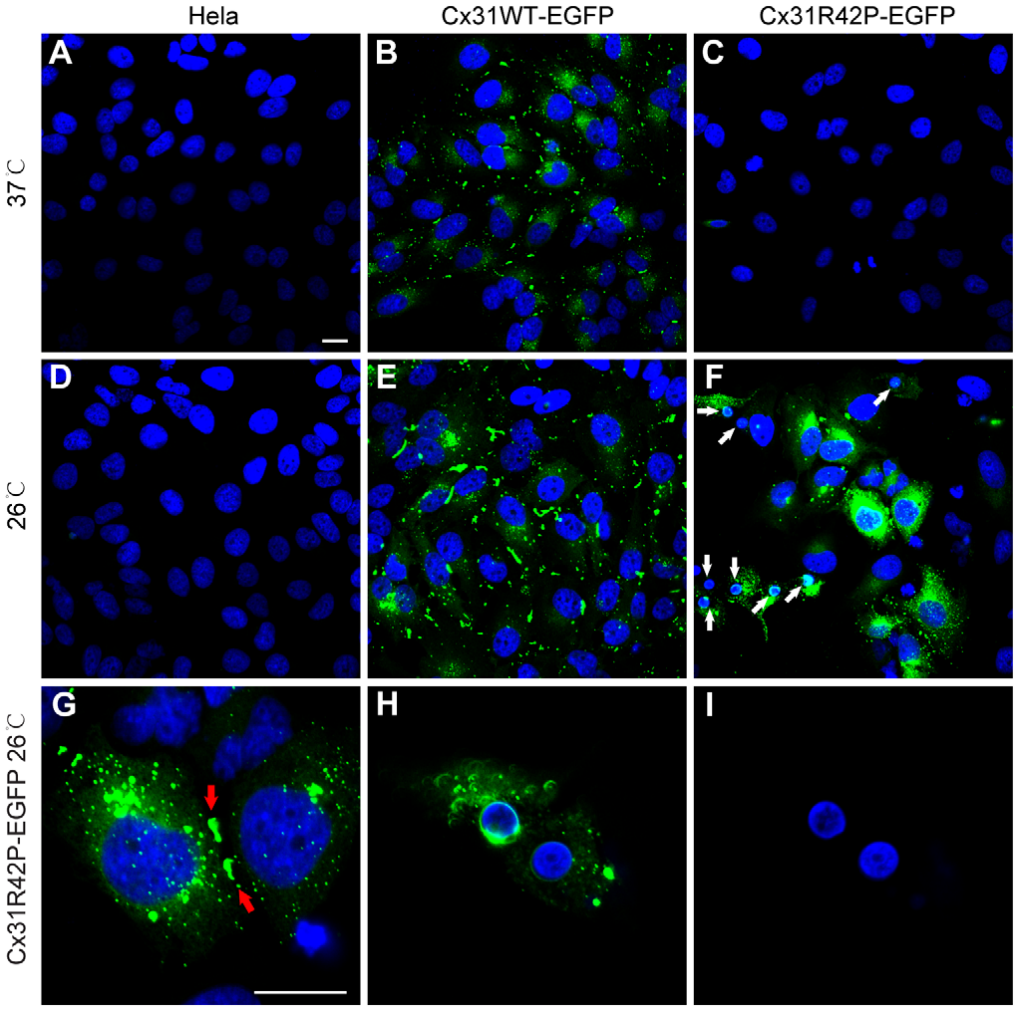
Intracellular distribution of Cx31 variants in stable cell lines. Hela cells stably expressing Cx31WT, Cx31R42P and their control line (Hela) were grown either at 37°C or at 26°C. Expression of Cx31 variants (green) and cell nuclei (blue) are shown. High magnification pictures of Cx31R42P-expressing cells are also shown to exemplify the gap junction plaque-like structures and small condensed nuclei (bottom panel). Note that Cx31WT-EGFP form gap junction plaques between adjacent cells incubated at 37°C (**B**). At 26°C, these structures are enlongated (**E**). Cx31R42P is barely detectable at 37°C (**C**). It is accumulated predominantly in cytoplasm (**F**, **G**) and can form gap junctions (**F** and **G**, red arrows) at 26°C. Small condensed nuclei (SN) are observed in Cx31R42P-expressing cells cultured at 26°C (**F**, **H and I**; white arrows). Bar = 20 µm.

We have recently found that EKV pathogenic Cx31 mutants are temperature sensitive mutants. In cells, mutant proteins are rapidly degraded at 37°C while they become stable and form functional gap junctions at 26°C (unpublished data). To further investigate the pathophysilogical function of EKV pathogenic mutants, we stably expressed Cx31WT and Cx31R42P in Hela cells. At 37°C, exogenous Cx31WT formed gap junction plaques between adjacent cells (Figure 1B). These plaques were further elongated at 26°C (Figure 1E). In contrast, Cx31R42P-EGFP was hardly detectable at 37°C (Figure 1C). However, the mutant protein not only showed increased detection but also formed gap junction plaque-like structures when cells were cultured at 26°C. Meanwhile, cytoplasmic aggregates of Cx31R42P were also detected (Figure 1F, G). Similar observation was made in cells expressing myc-tagged Cx31 variants (Figure S1).

**Figure 2:**
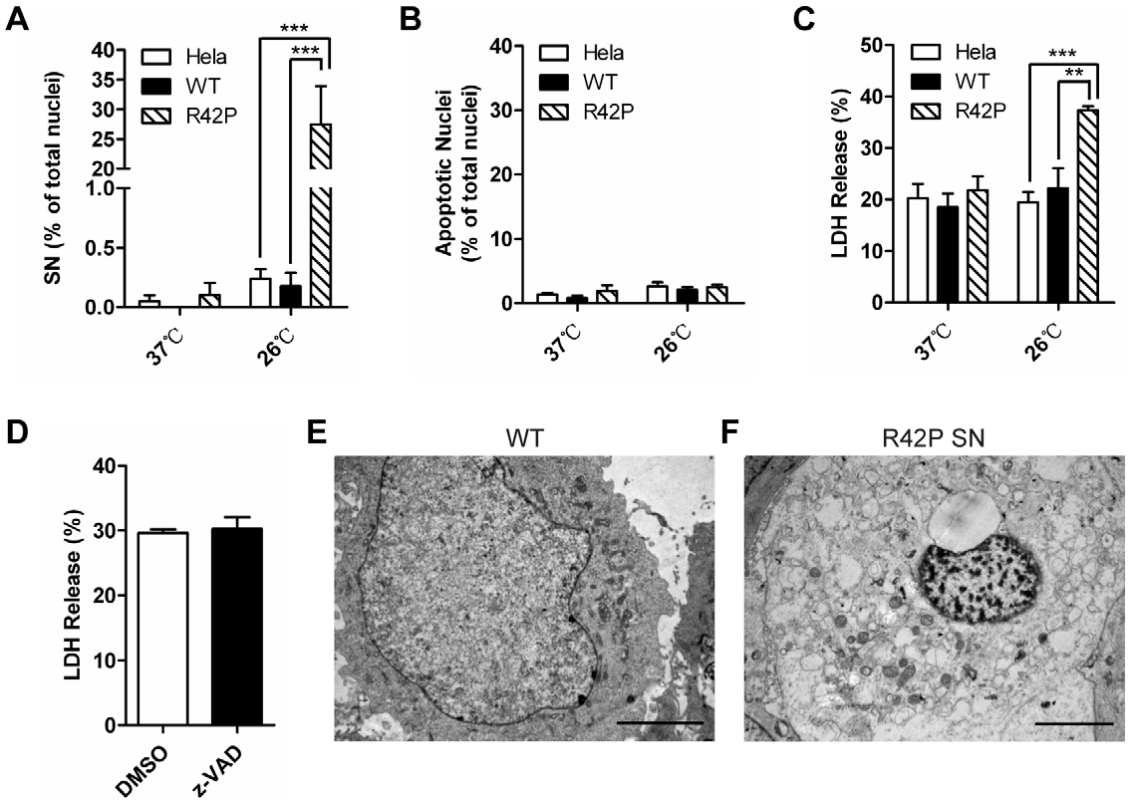
Expression of Cx31R42P induces necrotic cell death. Quantification of small condensed nuclei (SN) (**A**) and apoptotic nuclei (**B**) in Hela and Cx31WT- (WT) or Cx31R42P- (R42P) stable cells growing at 26°C or 37°C. The rate of LDH release in these cells are also shown (**C**). (**D**) The rate of LDH release in Cx31R42P cells with either pan-caspase inhibitor z-VAD treatment (20 µM) or control solvent (DMSO) treatment. Two stars: *P*<0.01; three stars: *P*<0.001. Error bars represent SEM. Representative TEM images of Cx31WT cells (**E**) and Cx31R42P cells with SN (**F**) are shown. Scale bars = 5 µm.

After 96 h of incubation at 26°C, a large portion of Cx31R42P-expressing cells exhibited condensed small nuclei (27.46±6.45%, N = 3; Figure 1F, H, I; Figure 2A; Figure S1F–I). Cells with the characterized small nuclei were positive with pepidium iodine (PI) staining (Figure S2). The LDH release from Cx31R42P cells was also significantly increased than that from Cx31WT cells and control Hela cells (Figure 2C). In contrast, few Cx31WT-expressing cells or control Hela cells were found to have small nuclei when they were cultured at either 26°C or 37°C (Figure 1 and 2A). Neither small nuclei phenotypes nor increased LDH release was found when Cx31R42P cells were cultured at 37°C (Figure 2A, C). Consistent with the observation, transient expression of Cx31R42P at 37°C resulted in similar small nuclei phenotype (Figure S3). The results suggest that expression of Cx31R42P cells induces cell death. Transmission electron microscopy (TEM) analysis revealed that Cx31R42P-induced small nuclei cells had morphological features of necrosis including translucent cytoplasm, swelling of organelles, disruption of the plasma membrane, dilatation of the nuclear membrane and condensation of chromatin into small, irregular patches [23] (Figure 2F). Moreover, the percentage of nuclei with typical apoptotic morphology was similar among cells expressing Cx31 variants and control Hela cells (Figure 2B), indicating that Cx31R42P unlikely induces apoptosis. Pan-caspase inhibitor z-VAD had little effect on Cx31R42P induced cell death (Figure 2D). Thus, overexpression of Cx31R42P likely results in necrotic cell death.

### Cx31R42P but not Cx31WT forms constitutively active hemichannels

**Figure 3:**
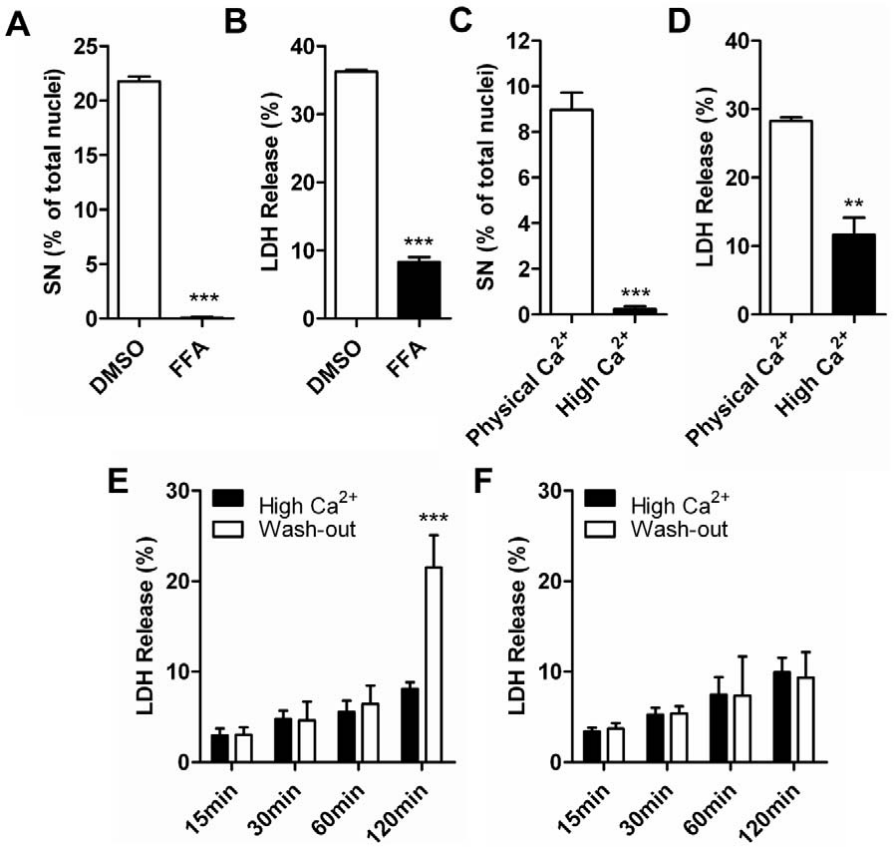
Suppression of Cx31R42P induced cell death by connexin hemichannel blocker and high extracellular calcium. (**A**) Quantification of Cx31R42P cells with SN after treatment with 200 µM FFA or control solvent DMSO. (**B**) The rate of LDH release of Cx31R42P cells after treatment with 200 µM FFA or control solvent DMSO. (**C**) Quantification of Cx31R42P cells with SN incubated at 26°C with physical or high Ca^2+^_o_. (**D**) The rate of LDH release of Cx31R42P cells incubated at 26°C with physical or high Ca^2+^_o_. (**E–F**) The rate of LDH release of Cx31R42P cells (**E**) and Cx31WT cells (**F**) at different time points after Ca^2+^_o_ treatment was washed out. Error bars represent SEM. Two stars: *P*<0.01; three stars: *P*<0.001.

To elucidate the molecular mechanism of EKV Cx31mutant induced cell death, we examined activity of gap junctions and hemichannels formed by Cx31R42P. Gap junctions formed by Cx31 variants effectively mediate dye transfer between cells (Figure S4). Therefore, the gap junction activity of Cx31R42P unlikely promotes cell death. FFA, a well-characterized connexin hemichannel blocker, significantly inhibited Cx31R42P induced cell death as evidenced by abolishing cells with small nuclei (Figure 3A) and significantly reducing LDH release (Figure 3B). FFA had little effect on Cx31WT-expressing cells (data not shown). The results suggest that Cx31R42P forms constitutively active hemichannels at cell surface and that hemichannel activity plays important roles in Cx31R42P induced cell death.

Extracellular Ca^2+^ (Ca^2+^_o_) regulates gating of connexin hemichannels [3], [24]–[25]. Connexin hemichannels are at low open probability at physiological Ca^2+^_o_ concentration and can further be inhibited with high Ca^2+^_o_ concentration [26]–[27]. We next examined whether Cx31R42P induced cell death is regulated by Ca^2+^_o_. High Ca^2+^_o_ was achieved by adding 3.2 mM calcium chloride to the culture medium to increase final Ca^2+^_o_ concentration to 5 mM. Consistent with a notion that Cx31R42P forms constitutive active hemichannels, Cx31R42P-induced cell death with small nuclei was completely inhibited by high Ca^2+^_o_ incubation (Figure 3C). The LDH release from cells expressing Cx31R42P was also significantly reduced (Figure 3D). In contrast, the percentage of typical apoptotic cell death was unaffected at high Ca^2+^_o_ (data not shown). Also, expression level of Cx31R42P remains similar in physiological and high Ca^2+^_o_ condition (Figure S5), indicating that the effect of high Ca^2+^_o_ is not resulted by reduced expression of mutant protein.

Furthermore, we incubated Cx31R42P cells in high Ca^2+^_o_ condition at 26°C for 4 days followed by changing into fresh medium with or without additional calcium. LDH release was measured at different time points after medium change. Significant increase in LDH release was observed in cells stably expressing Cx31R42P (Figure 3E) but not in cells stably expressing Cx31WT (Figure 3F) after cells were incubated in medium with physical Ca^2+^_o_ condition for 2 h. These results suggest that Cx31R42P induced cell death is Ca^2+^_o_ concentration dependent. Together, these results suggest that Cx31R42P forms constitutively active hemichennels to promote cell death.

### Cx31R42P induces ROS production via ER stress

**Figure 4:**
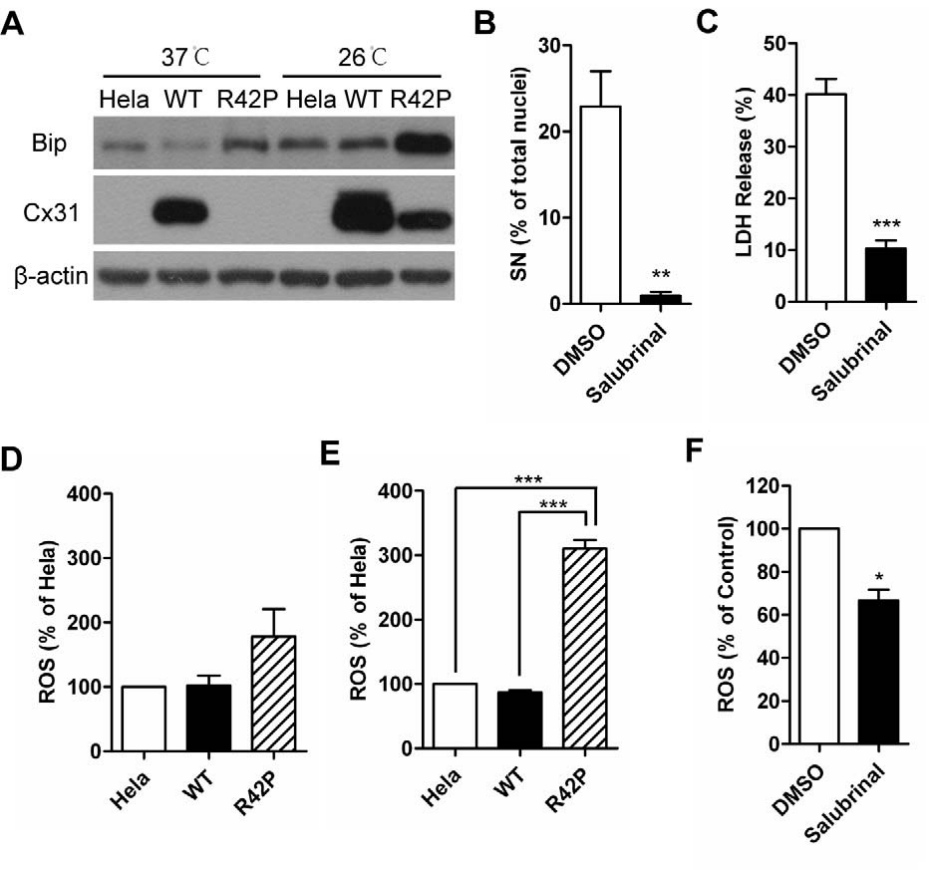
Cx31R42P induced ER stress promotes ROS production. (**A**) Bip expression in Hela, Cx31WT (WT) and Cx31R42P (R42P) cells incubated at 37°C or 26°C (upper panel). Expression of Cx31 variants (middle panel) and β-actin (lower panel) is shown. (**B**) Quantification of Cx31R42P cells with SN after salubrinal treatment (10 µM) or control solvent (DMSO) treatment. (**C**) The rate of LDH release in Cx31R42P cells with either salubrinal treatment (10 µM) or control solvent (DMSO) treatment. (**D–E**) Relative level of intracellular ROS in control Hela cells, cells stably expressing Cx31WT (WT) and Cx31R42P (R42P) when they are cultured at 37°C (**D**) or 26°C (**E**). (**F**) Relative ROS level in Cx31R42P cells with either salubrinal (10 µM) or control solvent (DMSO) treatment. Error bars represent SEM. One star: *P*<0.05; two stars: *P*<0.01; three stars: *P*<0.001.

A previous study indicates that EKV-pathogenic Cx31 mutants induce ER stress [21]. Consistent with the observation, expression of Cx31R42P, but not the Cx31WT, upregulated the ER stress marker Bip (Figure 4A). Treatment of salubrinal, an ER stress inhibitor [28], abolished cell death with small nuclei in Cx31R42P cells (Figure 4B). LDH release was also markedly decreased (10.27±1.57% v.s. 40.21±2.90%; N = 3) (Figure 4C). In contrast, cells with typical apoptotic nuclei remained unaffected (data not shown). The expression level of Cx31R42P was unchanged with the treatment (Figure S5). The results suggest that Cx31R42P induced ER stress plays important roles in regulating Cx31R42P cell death.

Overproduction of ROS contributes to the execution of necrosis [23], [29]–[30]. To investigate the mechanism of Cx31R42P induced cell death, we detected intracellular ROS production with 2′,7′-Dichlorofluorescin diacetate (DCFH-DA) staining followed by FACS analysis. Results showed that levels of ROS were similar among cells expressing Cx31 variants at 37°C (Figure 4D). At 26°C, the relative level of ROS in cells stably expressing Cx31R42P (310.20±13.63%, N = 3) was significantly higher than that in mock transfected cells (100%) and cells stably expressing Cx31WT (86.98±3.311%, N = 3) (Figure 4E). Interestingly, ROS production in Cx31R42P treated with ER stess inhibitor salubrinal was reduced to 66.66±5.094% comparing to that treated with solvent (Figure 4F). The results suggest that the overproduction of ROS in cells expressing Cx31R42P is, at least partially, caused by ER stress.

### ROS scavenger protects Cx31R42P-induced cell death

**Figure 5:**
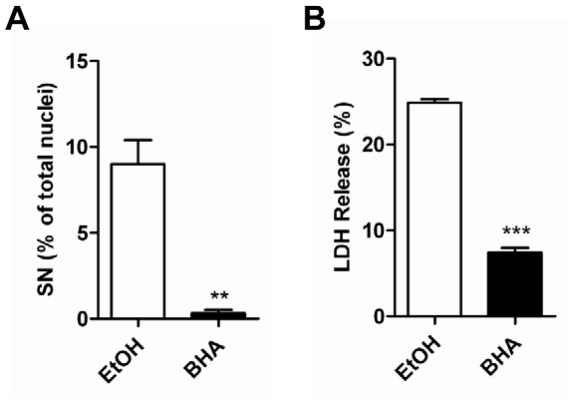
ROS scavenger rescues R42P induced cell death. (**A**) Quantification of Cx31R42P cells with SN with 100 µM BHA and control solvent ethanol (EtOH) treatment. (**B**) The rate of LDH release in Cx31R42P cells with 100 µM BHA and control solvent ethanol (EtOH) treatment. Error bars represent SEM. Two stars: *P*<0.01; three stars: *P*<0.001.

To determine whether ROS production contributes to Cx31R42P-induced cell death, we treated cells expressing Cx31R42P with ROS scavenger butylated hydroxyanisole (BHA). After four days of treatment, cell death were evaluated by DAPI staining and LDH release analysis. Results showed that the percentage of Cx31R42P cells with small nuclei was reduced to baseline (Figure 5A). Likewise, LDH release was significantly decreased in cells treated with BHA compared with those treated with solvent (7.45±0.53% v.s. 24.86±0.40%, N = 3) (Figure 5B). In contrast, cells with typical apoptotic nuclei appeared unaffected (data not shown) and the expression level of Cx31R42P remained unaffected (Figure S5). The results suggest that Cx31R42P induces necrotic cell death via a ROS mediated pathway.

### Gating of Cx31R42P hemichannels is regulated by the ROS scavenger

**Figure 6:**
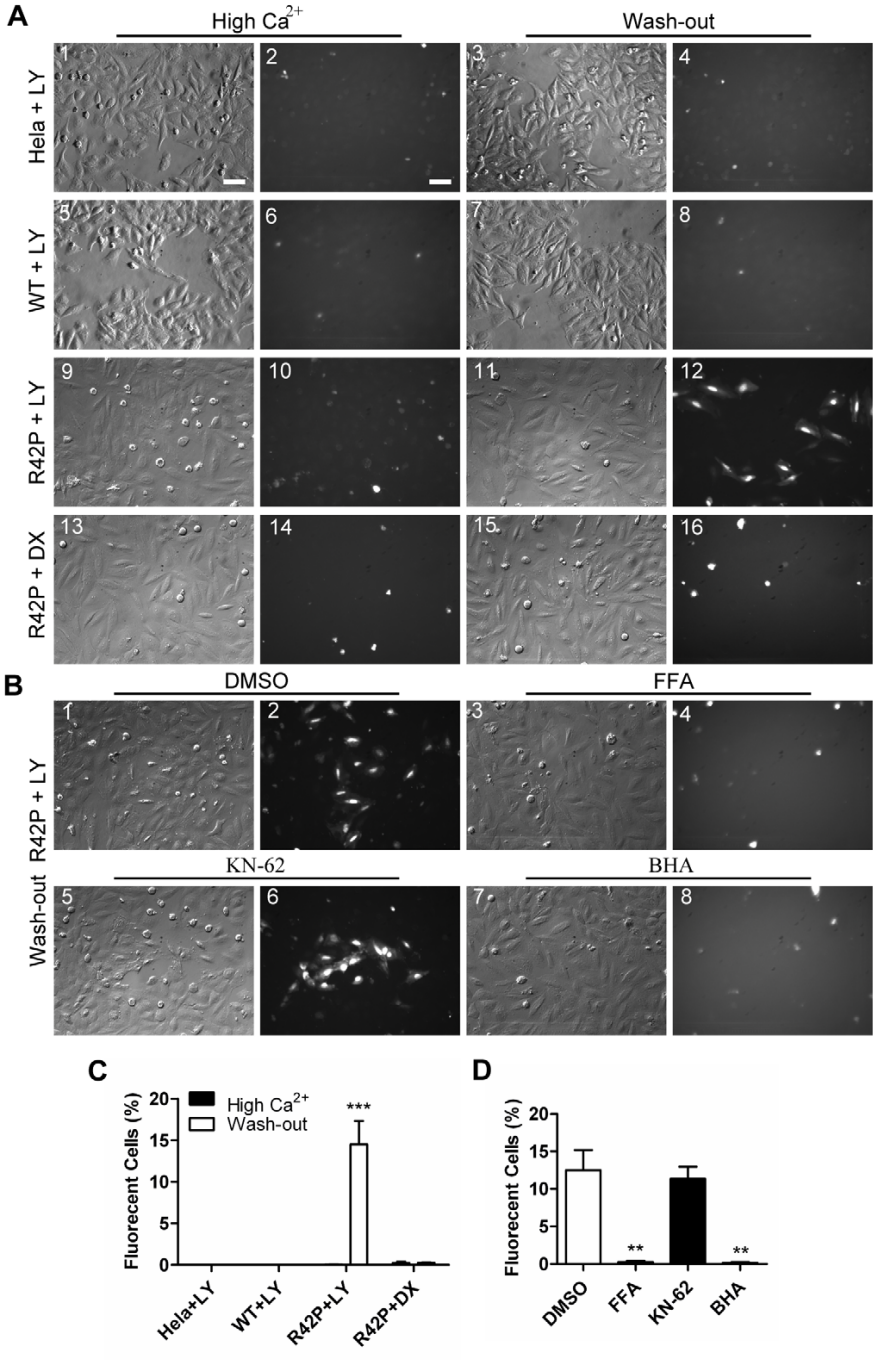
Cx31R42P mediates the lucifer yellow uptake. (**A**) Dye uptake with high Ca^2+^_o_ (High Ca^2+^) and 15 min after high Ca^2+^_o_ is washed (Wash-out). Images of Hoffman modulation contrast (A1, 3, 5, 7, 9, 11, 13, 15) and fluorescence (A2, 4, 6, 8, 10, 12, 14, 16) are shown. Cells tested include control Hela, Cx31WT (WT) and Cx31R42P (R42P) cells. Note that round dead cells were stained by both lucifer yellow (LY) and Dextran-FITC (DX). Hela and Cx31WT cells do not take up LY no matter high Ca^2+^_o_ treatment is washed out or not (1–8). Cx31R42P cells take up LY (11, 12) but not DX (15, 16) after high Ca^2+^_o_ is washed out. No LY uptake is observed in Cx31R42P stable cell line continuously to be incubated under high Ca^2+^_o_ condition (9, 10). (**B**) Pharmacological analysis of Cx31R42P hemichannels. Cells expressing Cx31R42P were pretreated with either control solvent DMSO, 200 µM connexin hemichannel blocker FFA, 10 µM P2X7 ATP receptor blocker KN-62, or 100 µM ROS scavenger BHA for 30 min. Note that FFA (3, 4) and BHA (7, 8) completely block LY uptake, but KN-62 shows little effect on LY uptake (5, 6). Scale bars = 20 µm. (**C**) Quantification of cells uptaking LY or DX. (**D**) Quantification of Cx31R42P cells uptaking LY after treatment with various pharmacological inhibitors. Error bars represent SEM. Two stars: *P*<0.01, three stars: *P*<0.001.

To examine the gating of Cx31R42P hemichannels, we assayed their uptake of dye with molecular weight less than 1 KD from the culture medium. Cells were cultured in high Ca^2+^_o_ containing medium at 26°C for 4 days followed by in fresh medium containing 0.5 mg/ml lucifer yellow (LY, MW: 457.25) or 10 mg/ml FITC labeled Dextran (DX, MW: 3–5KD) with or without additional calcium added. Fluorescent cells were counted after 15 min of incubation. Cells stained with both LY and DX are indicative of cell death while cells stained with LY but not DX are indicative of dye uptake through hemichannels. Under high Ca^2+^_o_ condition, few Cx31R42P cells were stained with either DX (Figure 6A13–14; C) or LY (Figure 6A9–10; C). Nevertheless, 14.51±2.822% of Cx31R42P cells were detected LY positive (Figure 6A11–12; C) while few of them was DX positive (Figure 6A15–16; C) under physical Ca^2+^_o_ condition. In contrast, both control Hela cells and Cx31WT cells were not stained with LY regardless of Ca^2+^_o_ concentration (Figure 6A1–8; C). The results suggest that Cx31R42P hemichannels, but not Cx31WT hemichannels, are constitutively open at physiological Ca^2+^_o_ concentration. Consistent with this notion, Cx31R42P cells were unable to take LY with treatment of a hemichannel inhibitor FFA (Figure 6B3–4; D). As a control, KN-62, a P2X7 ATP receptor inhibitor, showed no effect on LY uptake by the Cx31R42P cells (Figure 6B5–6; D). Interestingly, ROS scavenger BHA completely suppressed LY uptake by the Cx31R42P cells (Figure 6B7–8; D). The results suggest that ROS plays an important role in maintaining activity of Cx31R42P hemichannels.

### Cx31R42P hemichannels regulate ATP release from cells

**Figure 7:**
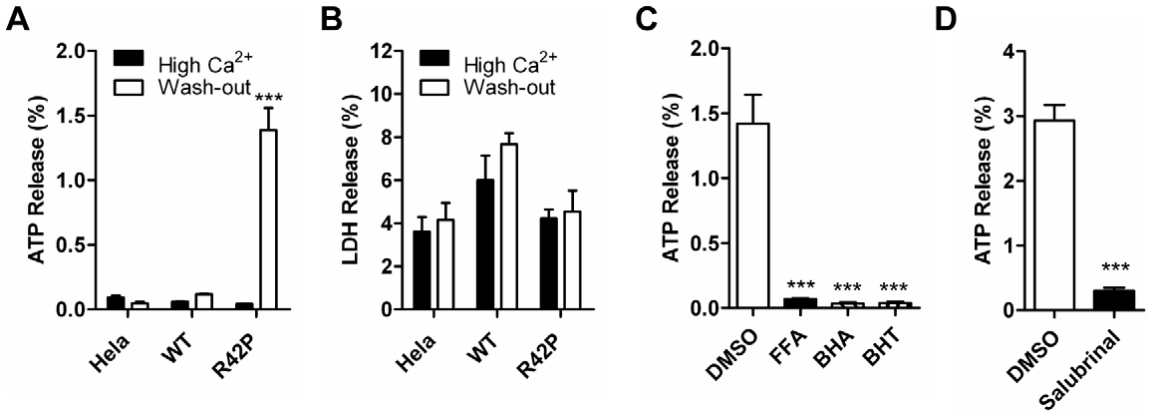
Cx31R42P hemichannels mediate ATP release. Cells were maintained in the medium with high Ca^2+^ (high Ca^2+^) and high Ca^2+^ depletion (Wash-out). The rate of ATP (**A**) and LDH release (**B**) from control Hela, Cx31WT (WT) and Cx31R42P (R42P) cells. (**C**) The rate of ATP release in Cx31R42P cells treated with 200 µM connexin hemichannel blocker FFA, 100 µM ROS scavenger BHA or BHT. (**D**) The rate of ATP release in Cx31R42P cells treated with 10 µM ER stress inhibitor salurinal or solvent DMSO. Error bars represent SEM. Three stars: *P*<0.001.

ATP has been recognized as an important autocrine and paracrine signaling molecule. In skin, ATP regulates keratinocyte differentiation and increase proliferation [31]–[32]. Connexin hemichannels mediates ATP release under various physiological and pathological conditions [2], [33]–[34]. To investigate whether Cx31R42P hemichannels regulate ATP release, cells were maintained under high Ca^2+^_o_ condition at 26°C for 4 days to induce Cx31R42P expression followed by maintaining cells in fresh medium with or without additional calcium. 15 min after medium change, level of ATP in Hela or Cx31WT cell culture medium remained similar under physical and high Ca^2+^_o_ condition (Figure 7A). In contrast, the rate of ATP release from Cx31R42P cells was significantly higher incubated in medium with physiological Ca^2+^ than that incubated in medium with high Ca^2+^(1.39±0.17% v.s. 0.04±0.37×10^−2^%, N = 3). Nevertheless, LDH level was not affected, suggesting that increased ATP release from Cx31R42P cells is not resulted by the plasma membrane leakage (Figure 7B). Furthermore, ATP release from Cx31R42P cells was completely blocked by hemichannel blocker FFA, ROS scavengers BHA and BHT (Figure 7C). Consistent with the result that ER stress inhibitor, salubrinal, reduced ROS production, it also reduced ATP release though Cx31R42P hemichannels (Figure 7D). The results suggest that Cx31R42P hemichannels mediate ATP release under physical Ca^2+^_o_.

## Discussion

In the current study, we report cell death upon expression of EKV pathogenic Cx31R42P. Cx31R42P induced cell death show the morphological characteristics of necrosis but not apoptosis. In addition, a pan-caspase inhibitor has little effect on Cx31R42P induced cell death. Our findings are in agreement with previous reports that expression of EKV pathogenic mutants induces cell death [17], [21]–[22]. In addition, we further defined the nature and molecular mechanisms of pathogenic Cx31 induced cell death.

Cx31R42P induced cell death is likely a result of activation of Cx31R42P hemichannels. Consistent with this notion, Cx31R42P induced cell death is greatly suppressed by either connexin hemichnannel inhibitor or by increasing extracellular calcium concentration. It is well established that high extracellular calcium inhibits gating probability of connexin hemichannels [3], [6], [25]–[26].

Cx31R42P is considered mostly unfolded protein. Its unstable nature at 37°C and formation of intracellular aggregates in 26°C further support this notion. Both EKV and hearing loss pathogenic Cx31 mutants are reported to induce ER stress [19], [21]. Thus, it is not surprise that expression of Cx31R42P causes ER stress, therefore, ROS overproduction [35]–[37]. Accordingly, inhibition of ER stress suppresses ROS production in cells expressing Cx31R42P. To our surprise, ROS scavenger treatment not only prevents Cx31R42P-induced cell death but also inhibit the activity of Cx31R42P hemichannels. Thus, the results suggest that the excessive ROS likely results in open of Cx31R42P hemichannels. This creates a feed-back mechanism to further enhance the deleterious of Cx31R42P mutant protein. It remains unclear how the ROS will regulates the gating of Cx31R42P hemichannels. Nevertheless, we cannot exclude the direct involvement of Cx31R42P induced ROS in executing cell death.

EKV is a rare hereditary skin disease pathologically characterized by hyperkaretostic perikaretosis [17], [38]. Both abnormal differentiation and hyperproliferation of karetinocytes are detected in the lesion. It remains unclear how the mutant induced cell death to contribute the disease initiation and progression. One potential mechanism is that cell death promotes inflammation that produces cell growth-prone cytokines. Consistent with this hypothesis, inflammation is often detected in the disease tissues of EKV patients [17], [38]–[40].

## Materials and Methods

### 

#### Materials

Hela cells were obtained from ATCC and maintained as suggested by the provider. The EGFP antibody was purchased from Clontech. Myc-tag antibody was purchased from Cell Signaling. All secondary antibodies were purchased from Jackson ImmunoResearch. Salubrinal and z-VAD were purchased from Santa Cruz and Promega respectively. All other reagents were from Sigma.

EGFP-tagged and myc-tagged cDNAs encoding Cx31 variants and Hela cells stably expressing Cx31 variants were generated as described previously [19], [41]. The stable cell lines were maintained in 200 µg/ml G418. To induce Cx31R42P expression, cells were seeded at 10% confluence, cultured at 37°C for four days followed by 26°C for four days.

#### Immuno-assays

Immunofluorescent staining and immunoblotting were done essentially as described previously [42].

#### Quantification of cell death

Cells were fixed in 4% paraformaldehyde for 10 min followed by being penetrated with 0.1% tritonX-100, and then the nuclei were stained with 1 µg/ml DAPI. The pictures were randomly taken with a fluorescence microscopy (Leica DMI 3000B). Over 500 cells were counted for each condition. Each experiment was repeated for three times.

#### LDH release detection

 Cell culture medium was collected and centrifuged at 5000×*g* for 10 min to remove the detached dead cells and cell debris. Cells were lysed in a lysis buffer (0.1% triton X-100 in PBS) for 10 min at 4°C. LDH in the medium and cell lysates were measured using CytoTox 96® Non-Radioactive Cytotoxicity Assay (Promega) according to manufacture's instructions. The rate of LDH release was given by: %LDH release = medium A492/total A492 = medium A492/medium A492+ cell lysate A492. Each experiment was repeated for three times.

#### TEM

Cells were trypsinized, pelleted by centrifugation, fixed in 2.5% glutaric dialdehyde and postfix treated with 2% osmium tetroxide. The samples were dehydrated with a graded series of acetones, infiltrated in acetone/resin (1∶1) and embedded in Epon. Fifty nanometer sections were collected and double stained with uranyl acetate and lead nitrate. The samples were examined with a transmission electron microscope (JEOL-1230) at 80 Kv accelerating voltage.

#### ROS detection

Cells were digested with collagenase IV (Gibco), pelleted and suspended in the medium containing 20 µM DCFH-DA. After 30 min of incubation, cells were centrifuged at 2000×*g* for 10 min, resuspended in a fresh medium and subjected to FACS analysis (Beckman-Coulter MoFLo XDP cell sorter). The results were analyzed with FlowJo 7.6 software for the mean fluorescence intensity (MFI). The data presented are the values relative to those obtained in Hela cells or solvent-treated control. The experiments were repeated for three times.

#### Dye uptake experiments

The dyes (0.5 mg/ml lucifer yellow and 10 mg/ml Dextran-FITC) were loaded to cells in the fresh medium with or without additional 3.2 mM calcium chloride. Fifteen minutes later, dye was removed. Cells were washed three times with medium containing additional 3.2 mM calcium chloride. Pictures were randomly taken using fluorescence microscopy Leica DMI 3000B. Over 500 cells were analyzed for each condition. The experiments were repeated for three times.

#### Detection of ATP release

Cell culture medium was collected and centrifuged at 5000×*g* for 5 min to remove the detached dead cells and cell debris. ATP in the medium and cells was measured using a CellTiter-Glo® Luminescent Cell Viability Assay (Promega) according to manufacture's instructions. The rate of ATP release was given by: %ATP release = medium RLU/total RLU = medium RLU/medium RLU+ cells RLU.

#### Statistic analysis

Statistic analyses were performed using Prism 5 software (GraphPad). The data is presented as mean ± SEM. Two-tailed Student's *t* test was used to determine the significance of difference between 2 groups. One-way ANOVA with Tukey's and Dunnett's Multiple Comparison Test were used to assess the difference among three cell lines and between treatments groups against their respective controls, respectively. Two-way ANOVA with Bonferroni post-test was used to analyze the data of multiple-group treatments.

## Supporting Information

Figure S1 **Intracellular distribution of Cx31 variants in stable cell lines.** Hela cells stably expressing myc-tagged Cx31WT, Cx31R42P and their control line (Hela) were grown either at 37°C or at 26°C. Cells were immunostained with anti-myc (red) antibody and nuclei were stained with DAPI (blue). High magnification pictures of Cx31R42P-expressing cells are also shown to exemplify the gap junction plaque-like structures and small condensed nuclei (bottom panel). Note that Cx31WT-myc form gap junction plaques between adjacent cells incubated at 37°C (B) or at 26°C (E). Cx31R42P-myc is barely detectable at 37°C (C). It is accumulated predominantly in cytoplasm (F, G) and can form gap junctions (F and G, green arrow) at 26°C. Small condensed nuclei (SN) are observed in Cx31R42P-expressing cells cultured at 26°C (F and G, white arrows; H and I). Bar = 20 µm.
(TIF)

Figure S2 **Cells expressing Cx31R42P with condensed small nuclei are positive with PI staining.** Hela cells stably expressing Cx31WT, Cx31R42P and their control line (Hela) were grown at 26°C. Expression of Cx31 variants (green) and cell nuclei stained with Hochest 33258 (blue) are shown. Note that cells expressing Cx31R42P with small nuclei take up PI (red) (**G**, **H** and **I**). There are neither cells with small nuclei nor PI uptake in cells expressing Cx31WT (**D**, **E** and **F**) and control Hela cells (**A**, **B** and **C**). Bar = 20 µm.
(TIF)

Figure S3 **Cx31R42P induces cell death with small condensed nuclei when transient transfected into Hela cells.** EGFP-tagged cDNAs encoding Cx31 variants and EGFP-N1 vector were transfected into Hela cells. After transfection, the cells were maintained at 37°C for 48 h. Expression of Cx31 variants, EGFP (green) and cell nuclei (blue) are shown. High magnification pictures of cells transfected with cDNAs encoding Cx31R42P are also shown to exemplify the small condensed nuclei (bottom panel). Note that small condensed nuclei (SN) are observed in cells transiently expressing Cx31R42P at 37°C (**E** and **F**, white arrows; **G** and **H**). There are no SN in cells transiently expressing Cx31WT (**C** and **D**) or EGFP (**A** and **B**) at 37°C. Bar = 20 µm.
(TIF)

Figure S4 **Dye transfer in cells expressing Cx31WT and Cx31R42P.** Cells stably expressing myc tagged Cx31 variants were grown at 26°C. The donor cells were washed once with 0.3 M glucose and loaded with 10 µM 1,1′-dioctadecyl-3,3,3′,3′-tetramethylindocarbocyanine perchlorate (Dil) (Molecular Probes), 5 µM calcein AM (Molecular Probes) diluted in 0.3 M glucose at 37°C for 30 min. After trypsinized, the donor cells were washed once with culture medium, resuspended and added to recipient cells at a ratio of 1∶100 (donors: recipients). After 3 h of co-culture at 26°C, the cells were imaged using fluorescence microscopy Leica DMI 3000B. Images of Hoffman modulation contrast (HMC) (**A**, **D** and **G**) and fluorescence are shown. Donor cells were marked by Dil (red). Calcein-AM (green) transfer from donor cells to adjacent recipient cells is observed in Cx31WT cells (**D**, **E** and **F**) and Cx31R42P cells (**G**, **H** and **I**). No dye transfer is observed in control Hela cells (**A**, **B** and **C**). Red and white arrows show donor and recipient cells respectively. Bar = 20 µm.
(TIF)

Figure S5 **Expression of Cx31R42P in cells stably expressing Cx31R42P subjected to pharmacological treatment.** Cx31R42P cells were treated with High Ca^2+^_o_ (CaCl_2_), BHA, Salubrinal and solvents (H_2_O or DMSO) at 26°C. The expression of Cx31R42P (R42P, upper panel) and β-actin (lower panel) are shown.
(TIF)
