# Supplementary figures and images for: Pathogenic Connexin-31 Forms Constitutively Active Hemichannels to Promote Necrotic Cell Death
Source: PLoS One. 2012 Feb 29;7(2):e32531. doi: 10.1371/journal.pone.0032531 (PMC3290583; doi:10.1371/journal.pone.0032531)

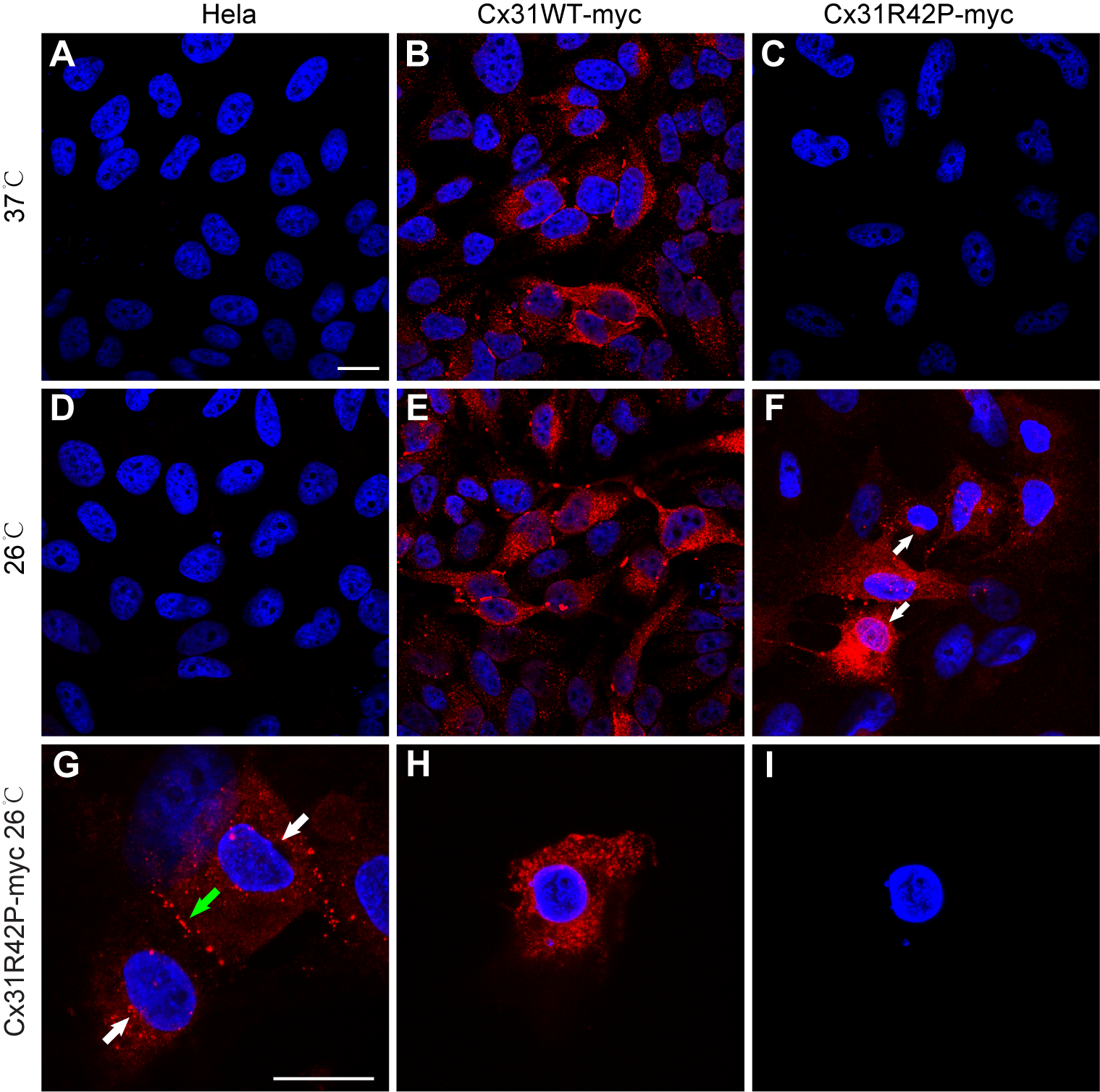

Supplement: Figure S1 — Intracellular distribution of Cx31 variants in stable cell lines. Hela cells stably expressing myc-tagged Cx31WT, Cx31R42P and their control line (Hela) were grown either at 37°C or at 26°C. Cells were immunostained with anti-myc (red) antibody and nuclei were stained with DAPI (blue). High magnification pictures of Cx31R42P-expressing cells are also shown to exemplify the gap junction plaque-like structures and small condensed nuclei (bottom panel). Note that Cx31WT-myc form gap junction plaques between adjacent cells incubated at 37°C (B) or at 26°C (E). Cx31R42P-myc is barely detectable at 37°C (C). It is accumulated predominantly in cytoplasm (F, G) and can form gap junctions (F and G, green arrow) at 26°C. Small condensed nuclei (SN) are observed in Cx31R42P-expressing cells cultured at 26°C (F and G, white arrows; H and I). Bar = 20 µm. (TIF) [file pone.0032531.s001.tif]

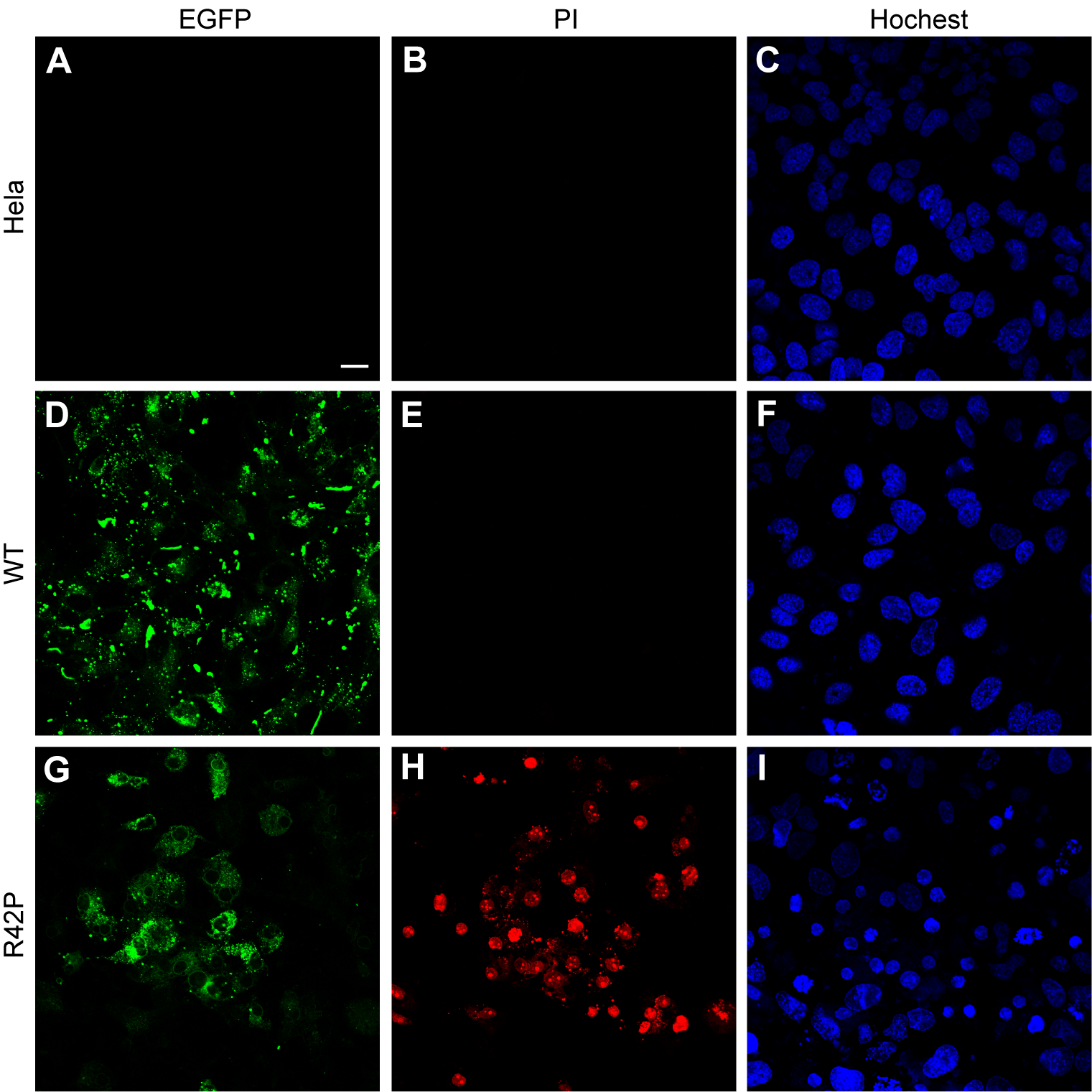

Supplement: Figure S2 — Cells expressing Cx31R42P with condensed small nuclei are positive with PI staining. Hela cells stably expressing Cx31WT, Cx31R42P and their control line (Hela) were grown at 26°C. Expression of Cx31 variants (green) and cell nuclei stained with Hochest 33258 (blue) are shown. Note that cells expressing Cx31R42P with small nuclei take up PI (red) (G, H and I). There are neither cells with small nuclei nor PI uptake in cells expressing Cx31WT (D, E and F) and control Hela cells (A, B and C). Bar = 20 µm. (TIF) [file pone.0032531.s002.tif]

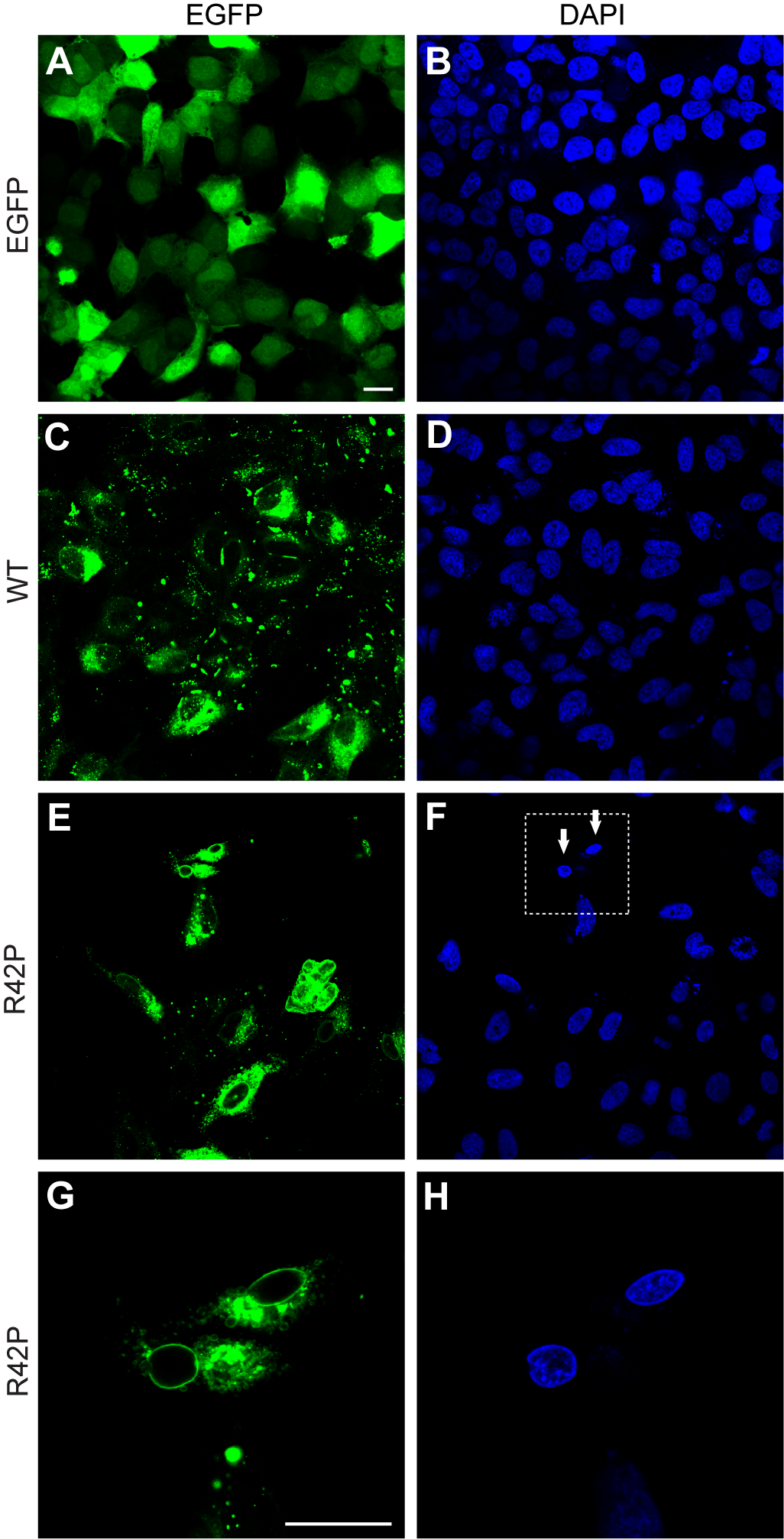

Supplement: Figure S3 — Cx31R42P induces cell death with small condensed nuclei when transient transfected into Hela cells. EGFP-tagged cDNAs encoding Cx31 variants and EGFP-N1 vector were transfected into Hela cells. After transfection, the cells were maintained at 37°C for 48 h. Expression of Cx31 variants, EGFP (green) and cell nuclei (blue) are shown. High magnification pictures of cells transfected with cDNAs encoding Cx31R42P are also shown to exemplify the small condensed nuclei (bottom panel). Note that small condensed nuclei (SN) are observed in cells transiently expressing Cx31R42P at 37°C (E and F, white arrows; G and H). There are no SN in cells transiently expressing Cx31WT (C and D) or EGFP (A and B) at 37°C. Bar = 20 µm. (TIF) [file pone.0032531.s003.tif]

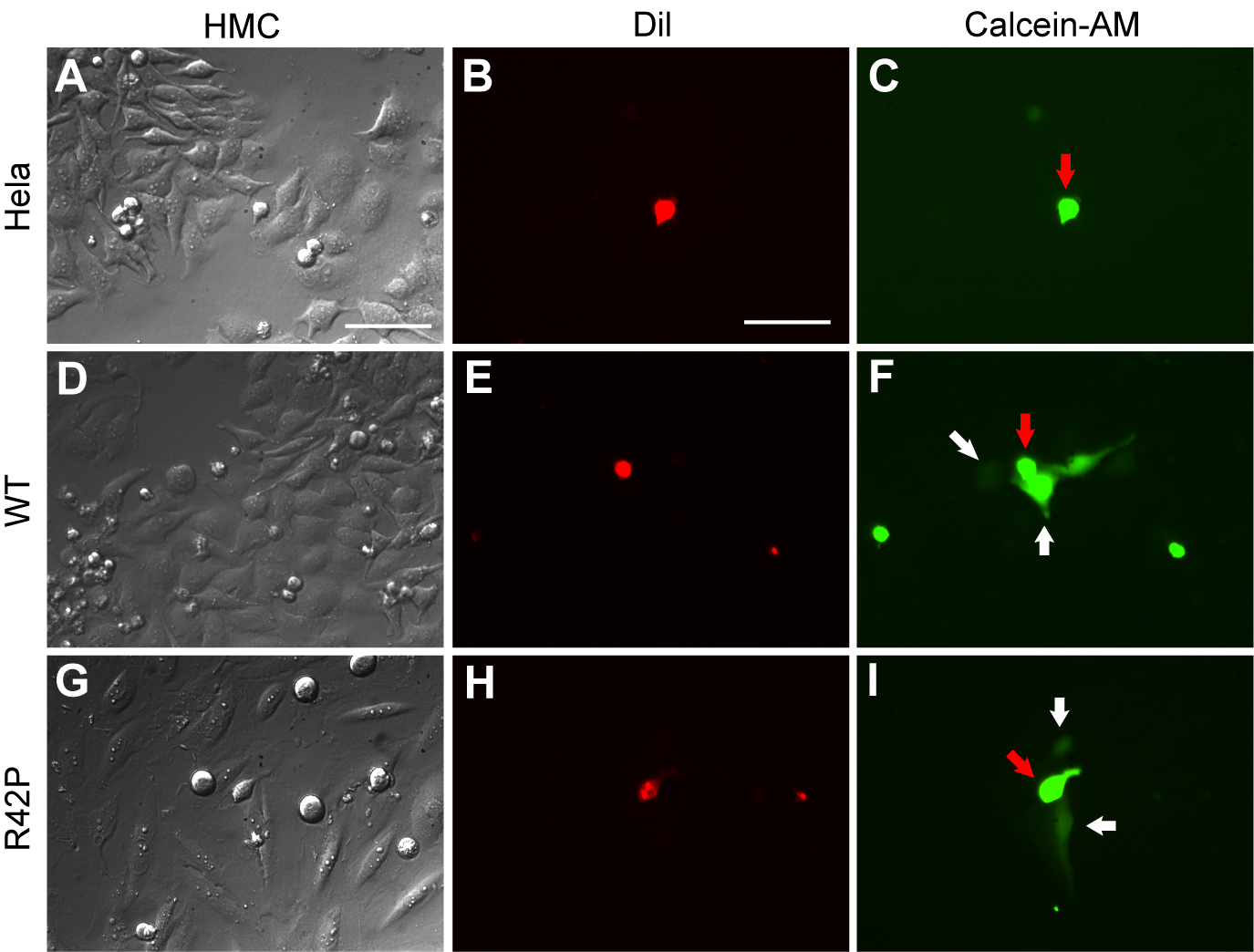

Supplement: Figure S4 — Dye transfer in cells expressing Cx31WT and Cx31R42P. Cells stably expressing myc tagged Cx31 variants were grown at 26°C. The donor cells were washed once with 0.3 M glucose and loaded with 10 µM 1,1′-dioctadecyl-3,3,3′,3′-tetramethylindocarbocyanine perchlorate (Dil) (Molecular Probes), 5 µM calcein AM (Molecular Probes) diluted in 0.3 M glucose at 37°C for 30 min. After trypsinized, the donor cells were washed once with culture medium, resuspended and added to recipient cells at a ratio of 1∶100 (donors: recipients). After 3 h of co-culture at 26°C, the cells were imaged using fluorescence microscopy Leica DMI 3000B. Images of Hoffman modulation contrast (HMC) (A, D and G) and fluorescence are shown. Donor cells were marked by Dil (red). Calcein-AM (green) transfer from donor cells to adjacent recipient cells is observed in Cx31WT cells (D, E and F) and Cx31R42P cells (G, H and I). No dye transfer is observed in control Hela cells (A, B and C). Red and white arrows show donor and recipient cells respectively. Bar = 20 µm. (TIF) [file pone.0032531.s004.tif]

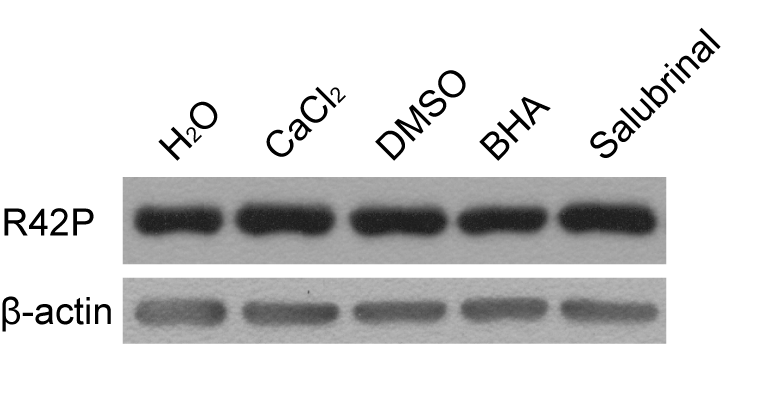

Supplement: Figure S5 — Expression of Cx31R42P in cells stably expressing Cx31R42P subjected to pharmacological treatment. Cx31R42P cells were treated with High Ca2+ o (CaCl2), BHA, Salubrinal and solvents (H2O or DMSO) at 26°C. The expression of Cx31R42P (R42P, upper panel) and β-actin (lower panel) are shown. (TIF) [file pone.0032531.s005.tif]
